# Baseline CD4^+^ and expansion of γδ T cells correlate with response to durvalumab in triple‐negative breast cancer patients

**DOI:** 10.1002/ctm2.1617

**Published:** 2024-04-25

**Authors:** Chiara Massa, Thomas Karn, Karsten Weber, Andreas Schneeweiss, Claus Hanusch, Jens Uwe Blohmer, Dirk‐Michael Zahm, Christian Jackisch, Marion van Mackelenbergh, Jörg Thomalla, Frederik Marmé, Jens Huober, Volkmar Müller, Christian Schem, Anja Müller, Elmar Stickeler, Katharina Biehl, Peter A. Fasching, Michael Untch, Sibylle Loibl, Carsten Denkert, Barbara Seliger

**Affiliations:** ^1^ Institute of Medical Immunology Martin Luther University Halle‐Wittenberg Halle Germany; ^2^ Institute for Translational Immunology Brandenburg Medical School Theodor Fontane Brandenburg an der Havel Germany; ^3^ Department of Obstetrics and Gynecology Goethe University Frankfurt Germany; ^4^ German Breast Group, GBG Forschungs GmbH Neu‐Isenburg Germany; ^5^ Nationales Centrum für Tumorerkrankungen Universitätsklinikum und Deutsches Krebsforschungszentrum Heidelberg Germany; ^6^ Rotkreuzklinikum München München Germany; ^7^ Gynäkologie mit Brustzentrum der Charite CCM Charité‐Universitätsmedizin Berlin Berlin Germany; ^8^ SRH Wald‐Klinikum Gera Gera Germany; ^9^ Department of Obstetrics and Gynecology Sana Klinikum Offenbach Offenbach Germany; ^10^ Klinik für Gynäkologie und Geburtshilfe Universitätsklinikum Schleswig‐Holstein Kiel Germany; ^11^ Praxis für Hämatologie und Onkologie Koblenz Germany; ^12^ Universitätsfrauenklinik Medizinische Fakultät Mannheim der Universität Heidelberg Heidelberg Germany; ^13^ Breast Cancer Cantonal Hospital St.Gallen St. Gallen Switzerland; ^14^ Department of Obstetrics and Gynecology Universitätsklinikum Hamburg‐Eppendorf Hamburg Germany; ^15^ Mammazentrum am Krankenhaus Jerusalem Hamburg Germany; ^16^ Klinik für Gynäkologie und Geburtsmedizin Uniklinik RWTH Aachen Aachen Germany; ^17^ Department of Obstetrics and Gynecology Universitätsklinikum Erlangen Erlangen Germany; ^18^ Department of Obstetrics and Gynecology HELIOS Klinikum Berlin Buch Berlin Germany; ^19^ Institute of Pathology Philipps‐University Marburg and University Hospital Marburg (UKGM) Marburg Germany

Dear Editor,

Immunomonitoring of patients with primary, non‐metastatic triple‐negative breast cancer (TNBC) from the GeparNuevo trial indicated that treatment with the immune checkpoint inhibitor (ICPi) durvalumab resulted in almost complete coverage of its target programmed death ligand 1 (PD‐L1) on circulating immune cells. Moreover, pathological complete response (pCR) upon the addition of durvalumab to neoadjuvant chemotherapy (NAC) correlated with higher pretreatment levels of CD4^+^ T cells and with expansion of γδ T cells during treatment.

Since TNBC lacks targetable disease drivers, but has a high lymphocytic infiltration, different attempts to implement immunotherapeutic approaches have been performed including ICPi, which improved clinical responses only in a limited number of patients thereby underlying the need to identify predictive biomarkers to better stratify patients for therapy.^1^

In this study, 63 TNBC patients of the window sub‐cohort of the randomized, double‐blind phase II GeparNuevo trial treated with NAC in the presence or absence of the anti‐PD‐L1 Ab durvalumab^2^ were analyzed. Blood was drawn at four different time points [i.e. T1 at baseline, T2 after the window treatment with durvalumab, T3 after nanoparticle‐bound paclitaxel (Nab‐Pac) and T4 at surgery after treatment with epirubicin and cyclophosphamide (EC); Figure S1] and characterized by multicolour flow cytometry using different Ab panels (Table S1). The baseline characteristics of TNBC patients undergoing immunomonitoring were not statistically different from those of the overall study (Table S2). However, the increase in pCR to durvalumab versus placebo in patients of the window sub‐cohort reached significance in the total trial,^2^ but not in the immunomonitored patients (64.7% vs. 58.6%, respectively, odds ratio [OR] = 1.29, 95% confidence interval 0.47–3.59, *p* = .620; Figure S2).

Characterization of the immune cell absolute counts, frequencies and phenotype during treatment highlighted many alterations (Table S3). The most consistent was the almost complete loss of PD‐L1 molecules on circulating cells already after the first application (Figure 1A). Since the Ab moiety of durvalumab has been engineered to avoid interaction with the complement and Fc receptors,^3^ this reduced detection is due to shielding of PD‐L1 and not due to the elimination of PD‐L1‐positive cells, as confirmed by an equal absolute number and frequency of T cells in durvalumab‐ and placebo‐treated patients (Figure S3).

**FIGURE 1:**
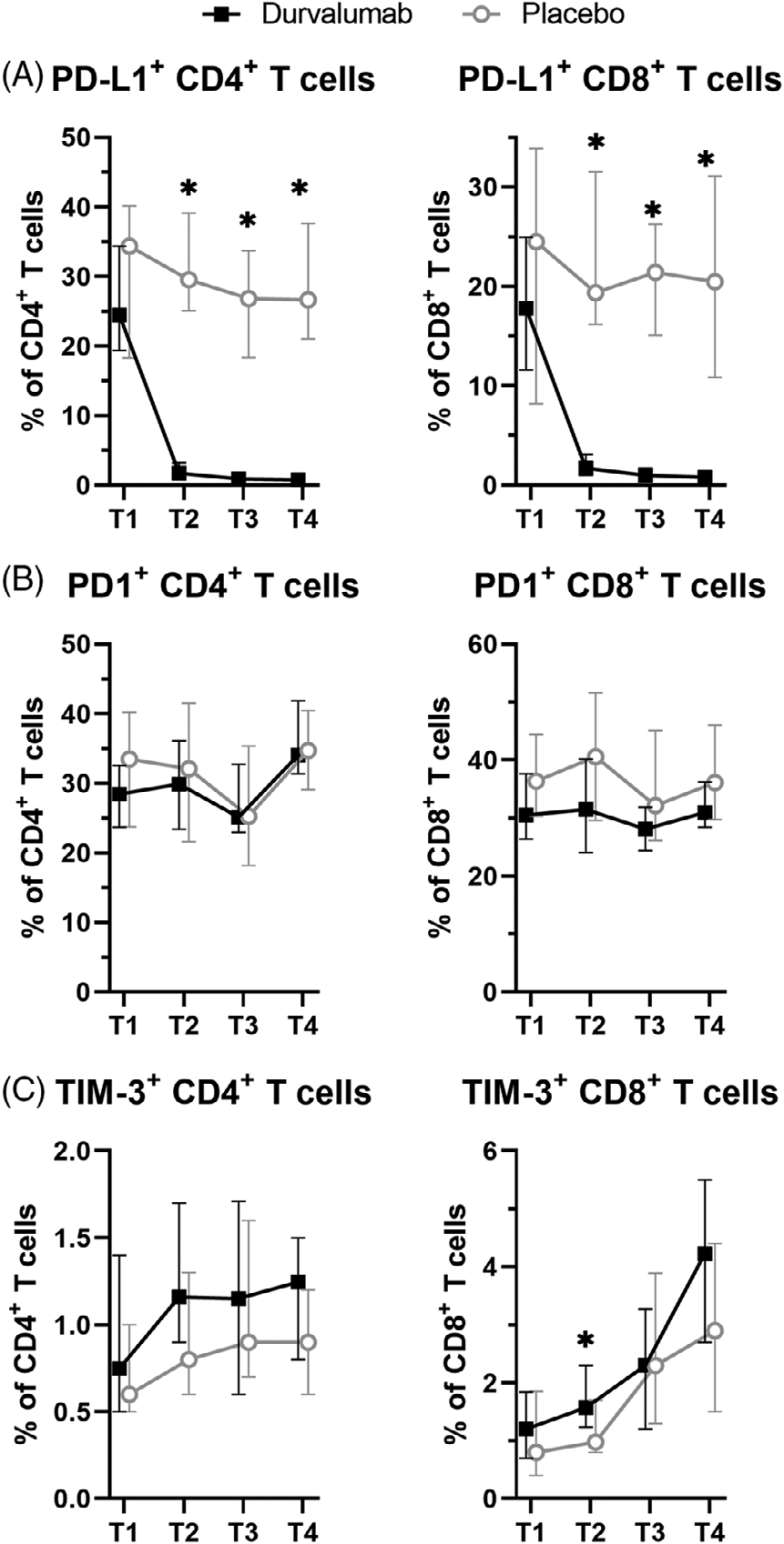
Biomarkers affected by durvalumab treatment. Frequencies of programmed death ligand 1 (PD‐L1) (A), PD1 (B) and TIM‐3 (C) expressing cells among CD4^+^ (*left*) and CD8^+^ T cells (*right*) along treatment. Shown are the median ± 95% CI of the monitored window patients at the different time points. *, *p* < .05 unpaired Wilcoxon test between durvalumab and placebo patients.

The only marginally more frequent pCR to durvalumab despite the homogenous loss of PD‐L1 availability might be due to different reasons. Despite NAC caused an upregulation of PD1 and TIM‐3 on T cells in the placebo arm of GeparNuevo,^4^ only a transient upregulation of TIM‐3 on CD8^+^ T cells was found after the window treatment with durvalumab (Figure 1B,C) thereby excluding the involvement of additional immune escape mechanisms in the failure to enhance pCR.^5^ An alternative explanation might be that the loss of the availability of PD‐L1 within the tumours is not as complete as in the periphery, as demonstrated in a murine setting.^6^

The search of predictive immune cell marker(s) for patients´ stratification indicated that high frequencies of CD4^+^ T cells at recruitment (T1) were associated with a benefit in the response rate to durvalumab treatment compared to placebo, while low frequencies were not (Figure 2, *top*). This result is in line with a report demonstrating that patients with tumour cells expressing high HLA‐DR levels, which therefore might be able to directly interact with CD4^+^ T cells, have a better response to durvalumab.^7^ Moreover, despite their OR values were not statistically significant, the interaction between the “high” and “low” marker groups with the treatment arm with respect to pCR was significant for the frequency of CD4^+^ T cells expressing HLA‐DR together with CD45RA or CCR7 as well as the frequency of CD8^+^ T cells expressing neither CD38 nor CCR7 (Figure 2, *bottom*). Figure S4 shows the values of these markers for each individual patient with respect to treatment arm and pCR.

**FIGURE 2:**
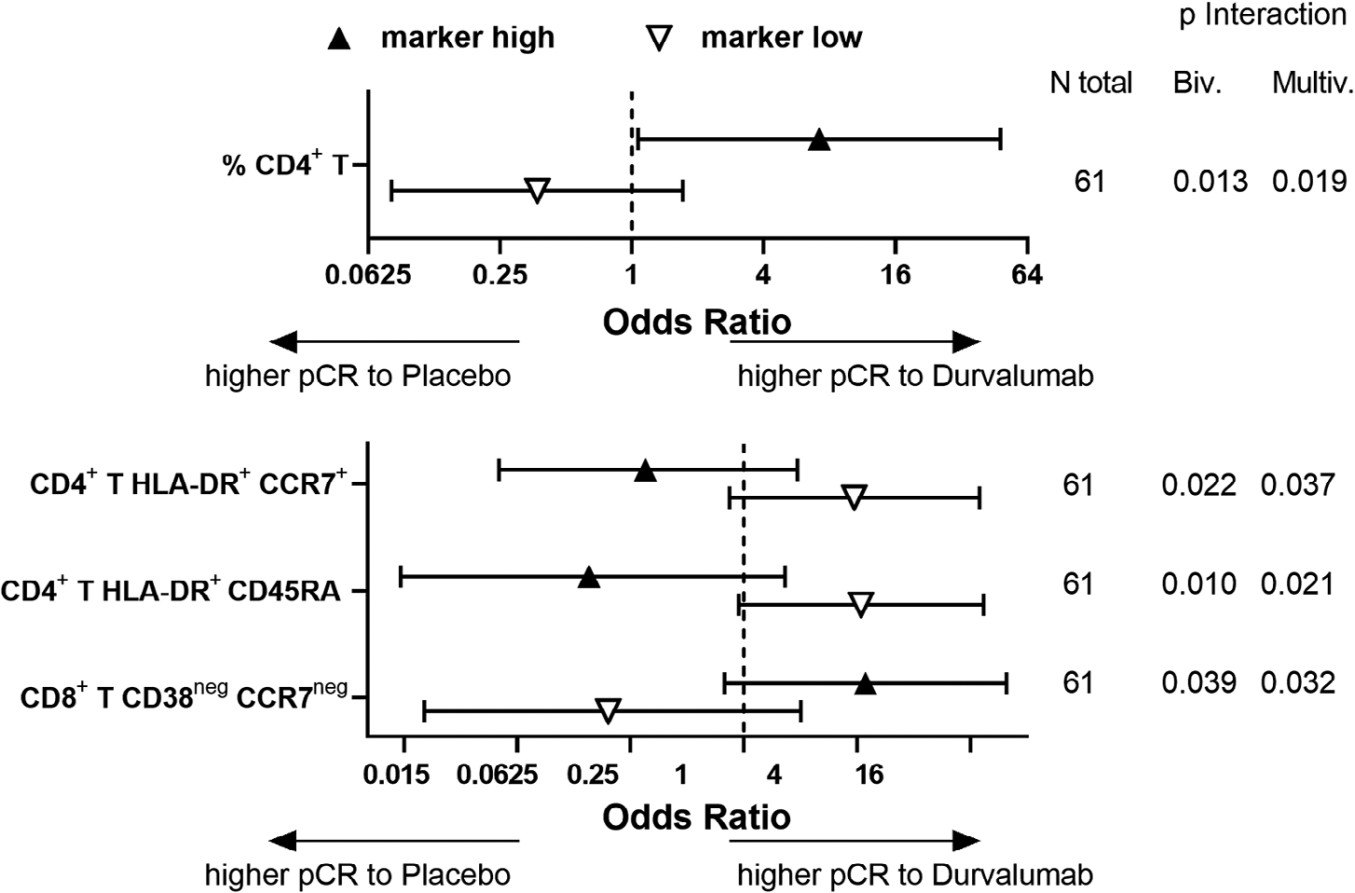
Biomarkers at recruitment predicting response to durvalumab treatment. Window patients were dichotomized into “marker high” and “marker low” groups for each marker at recruitment based on the median value for the complete GeparNuevo immune‐monitored cohort. Within each group, the odds ratios (ORs) for response to durvalumab over placebo were calculated. Wald tests for the interaction of the dichotomized marker and the treatment arm were also calculated from logistic regression models for pathological complete response (pCR): each “bivariable” interaction model was complemented by a “multivariable” model containing all predictive clinical baseline variables (significance level 0.2 in immune‐monitored patients), namely stromal tumour infiltrating cells (TIL, continuous variable) and grading (G2 vs. G3, no G1 tumours within the GeparNuevo trial). Among the markers reaching statistical significance for the interaction with treatment for pCR between the “high” and “low” marker groups, markers having also statistically significant OR in at least one of the two groups are provided in the top panel, the remaining markers in the bottom panel. The OR values for the different markers are shown together with the number of evaluated patients and the *p*‐value for the interaction in both the bi‐ and the multivariable evaluation.

To establish markers able to identify patients responding to durvalumab treatment, changes in immune cell composition during treatment were evaluated. Different parameters reached statistical significance at one time point (Figure 3 and Figures S5–S7), but there were two more constant results. Unexpectedly, a loss in the absolute number of different immune cell populations during treatment including peripheral blood mononuclear cells, B cells and total or CD8^+^ T cells significantly correlated with higher pCR in durvalumab‐treated patients (Figure 3 and Figures S5–S7). In addition, an increased pCR to durvalumab significantly correlated with an expansion of γδ T cells throughout the treatment (Figure 4), even if it reached statistical significance only after window and Nab‐Pac (Figure 3A,B and Figures S5 and S6) and not at surgery. Despite the focus of ICPi‐based therapies is mainly the release of CD8^+^ T cells from their exhausted state, also activated γδ T cells can express ICP molecules and thus recover their functionality upon blockade of the PD1/PD‐L1 axis. Since γδ T cells directly recognize tumour cells independent of HLA antigen presentation, they represent promising effector cells for cancer immunotherapy^8^ and those results might help improve their clinical implementation. Interestingly, at surgery, an increase in the subpopulation of CD16^+^ CD56^neg^ γδ T cells was associated with higher pCR rates to durvalumab (Figure 3C and Figure S7). Since CD16 expression on γδ T cells has been associated with an effector memory phenotype, while CD56 expression with their cytotoxic capacity,^9^ such enhanced frequencies of CD16^+^CD56^neg^ γδ T cells in the blood at surgery might be due to enhanced migration of the cytotoxic CD56^+^ population into the tumour with a consequent depletion from the periphery.

**FIGURE 3:**
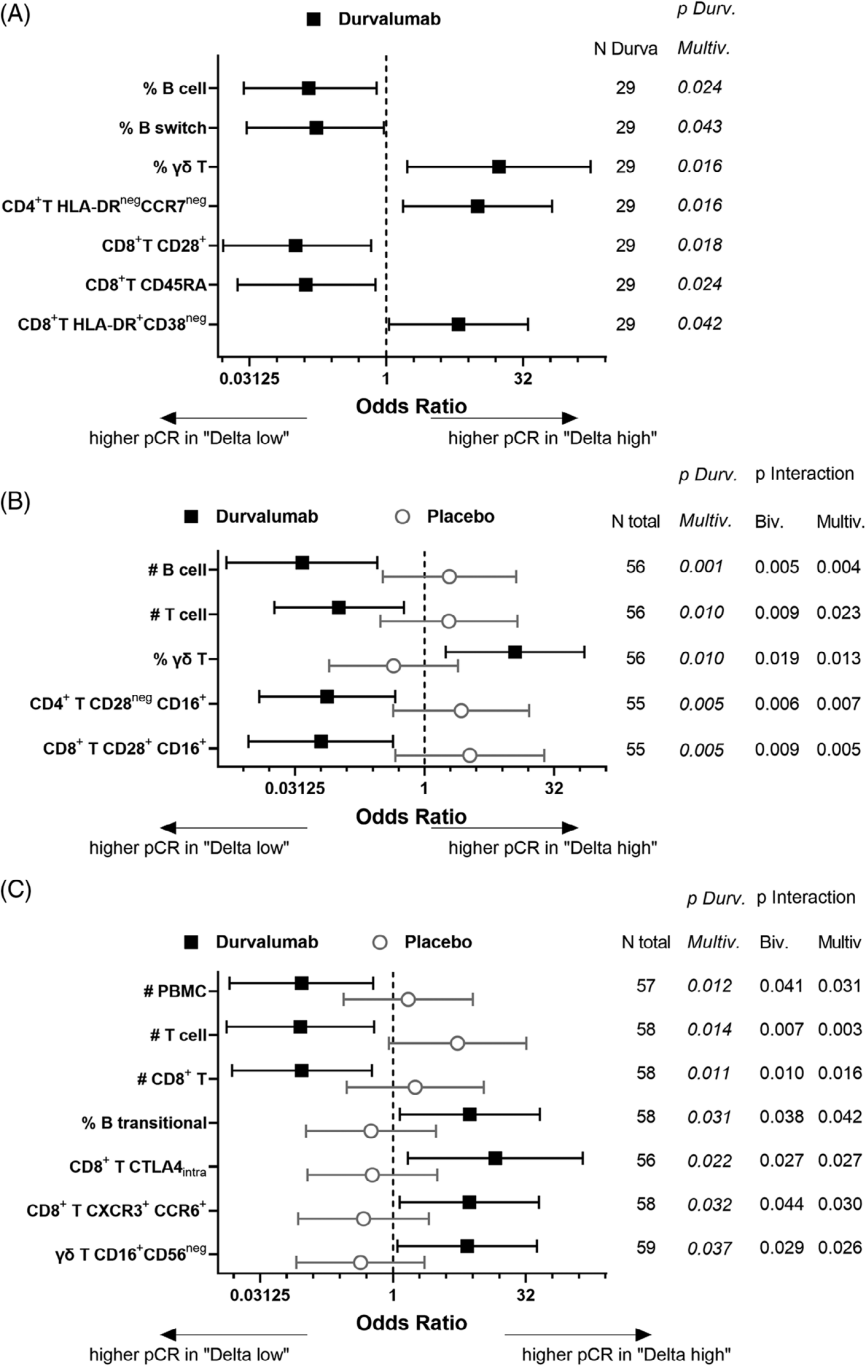
Changes of biomarkers along treatment in correlation to the clinical outcome. For each biomarker the changes between the different treatment time points (i.e. T2, T3 or T4) and recruitment (T1) were calculated as the ratio between the absolute cell counts [i.e. (Biomarker #_T2/T3/T4_ / Biomarker #_T1_)] or as deltas between the frequencies [i.e. (Biomarker %_T2/T3/T4_ – Biomarker %_T1_)]. The window patients from the durvalumab and from the placebo arm were then dichotomized into “Delta high” and “Delta low” based on the median of the changes within the complete immune‐monitored cohort of the GeparNuevo trial. The odds ratio (OR) and interaction values were calculated as described in the legend in Figure 2. (A) Markers, whose changes in durvalumab patients after the window treatment (i.e. T2–T1) have OR reaching statistical significance in the uni‐/bi‐variable (not shown) and multivariable logistic regression, are shown together with the number of patients that were evaluated and the *p*‐values. (B, C) Markers, whose changes after Nab‐Pac (i.e. T3–T1; B) or at surgery (i.e. T4–T1; C) have OR reaching statistical significance in the durvalumab arm as well as in the Wald interaction with the placebo arm, are shown together with the number of patients analyzed and the respective *p*‐values. With respect to the B cell subpopulations, “switch” are the CD27^+^ IgD^−^ switch memory cells, whereas “transitional” are the CD24^+^ CD38^+^ transitional cells among naïve (CD27^−^ IgD^+^) B cells.

**FIGURE 4:**
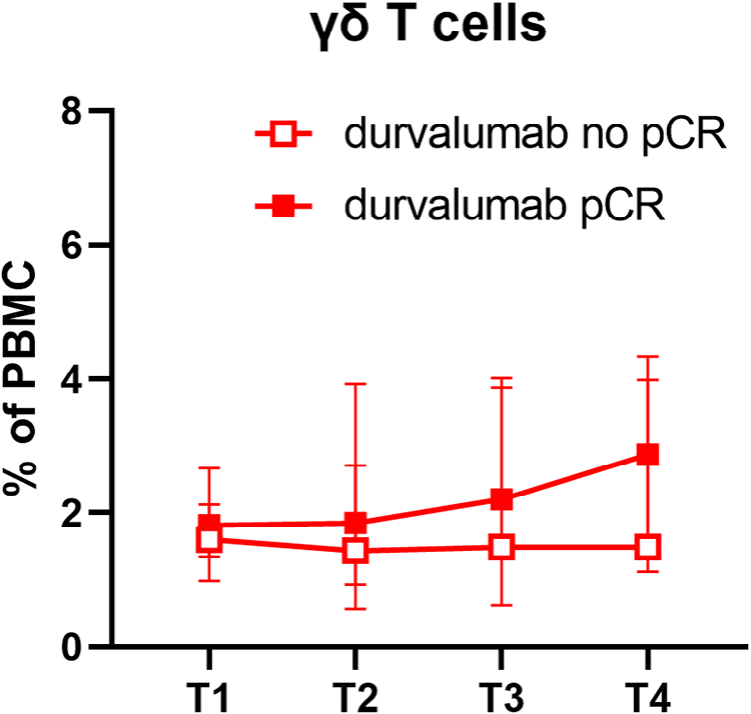
γδ T cell frequencies along treatment. Frequencies of γδ T cells among peripheral blood mononuclear cell (PBMC) in patients during treatment. Shown are the median together with the 95% confidence interval (CI) for patients in the durvalumab arm in relation to their clinical outcome.

Overall, the immunomonitoring results suggest that patients with a high total frequency of CD4^+^ T cells at recruitment have a higher probability of responding to the addition of durvalumab to NAC. Moreover, patients with an expansion of γδ T cells in the blood upon durvalumab treatment have a higher probability of response and should therefore continue with this therapy, whereas for patients without expansion alternative treatments have to be considered. However, these data have to be validated in larger cohorts of TBNC patients undergoing the same treatment regimen before their use as biomarkers for monitoring therapy response.

## AUTHOR CONTRIBUTIONS

Barbara Seliger and the coordinating committee (Thomas Karn, Sibylle Loibl and Carsten Denkert) designed the experiment; Anja Müller and Katharina Biehl performed the immunomonitoring staining; Chiara Massa analysed the data; Karsten Weber performed the statistical evaluation; Andreas Schneeweiss, Claus Hanusch, Jens‐Uwe Blohmer, Dirk‐Michael Zahm, Christian Jackisch, Marion van Mackelenbergh, Jörg Thomalla, Frederik Marmé, Jens Huober, Volkmar Müller, Christian Schem, Elmar Stickeler, Peter A. Fasching and Michael Untch provided the clinical samples; Chiara Massa and Barbara Seliger wrote the manuscript with feedback from Thomas Karn, Karsten Weber and Carsten Denkert; all the authors approved the manuscript.

## CONFLICT OF INTEREST STATEMENT

Thomas Karn, Andreas Schneeweiss, Claus Hanusch, Christian Jackisch, Jens Huober, Volkmar Müller, Christian Schem, Elmar Stickeler, Michael Untch, Sibylle Loibl and Barbara Seliger declare a possible conflict of interest.

## FUNDING INFORMATION

GBG Forschungs GmbH was the sponsor of the GeparNuevo trial, which was financially supported by AstraZeneca and Celgene. This translational research study was designed and conducted in cooperation with the GBG Forschungs GmbH and financially supported by Celgene, the German Cancer Aid (grant #70113450, CD, BS and SL) and the Mildred‐Scheel Stiftung (grant # 70113311, BS).

## ETHICS STATEMENT

The clinical trial was approved by the ethics committee of the Landerärztekammer in Hessen, Germany (# FF116/2015). All patients provided written informed consent for study conduct, biomaterial collection and analysis.

## Supporting information

Supporting Information

## Data Availability

The results of the immune‐monitoring are available from the corresponding author upon request. All relevant data are within the paper and its supporting information files. The data underlying the results presented in the study are available from GBG. Some restrictions apply due to the confidentiality of patient data. Interested groups may use the “Cooperation Proposal Form” at https://www.gbg.de/en/research/trafo.php. Data can be requested in the context of a translational research project by sending the form to trafo@gbg.de. Translational research proposals are approved by the GBG scientific boards.
