# Supplementary material for: Baseline CD4+ and expansion of γδ T cells correlate with response to durvalumab in triple‐negative breast cancer patients
Source: Clin Transl Med. 2024 Apr 25;14(4):e1617. doi: 10.1002/ctm2.1617 (PMC11045558; doi:10.1002/ctm2.1617)
Supplement: Supplementary file 1 — Supporting Information [file CTM2-14-e1617-s001.docx]

**Online only Material**

**Supplementary Table 1.** Ab panels used for blood phenotyping

**Supplementary Table 2.** Patients´ characteristics of the total and immune-monitored GeparNuevo trial

**Supplementary Table 3.** Blood markers changing differently along treatment between the durvalumab and the placebo arm of the GeparNuevo trial

**Supplementary Figure 1.** Scheme of the GeparNuevo trial

**Supplementary Figure 2.** Clinical outcome of the different sub-cohorts of the GeparNuevo trial

**Supplementary Figure 3**. Absolute cell counts and frequencies of CD4^+^ and CD8^+^ T cells during treatment

**Supplementary Figure 4.** Biomarkers at recruitment

**Supplementary Figure 5.** Biomarkers after the window treatment with durvalumab

**Supplementary Figure 6.** Biomarkers after Nab-Pac

**Supplementary Figure 7.** Biomarkers at surgery

**Supplementary Table 1.** Ab panels used for blood phenotyping and absolute cell count

|  | Fitc | Pe | PeCy5 | PeCy7 | APC | APC-H7 | BV421 | BV510 | BV605 |
| --- | --- | --- | --- | --- | --- | --- | --- | --- | --- |
| surf | γδ TCR | CD56 | CD4 | CD28 | CD16 | CD8 | CD45 |  | CD3 |
| surf | CD45 | CCR7 | CD4 | CD45RA | CD38 | CD8 |  | HLA-DR | CD3 |
| surf | slan | CD56 | CD123 | CD11c | CD16 | CD3+19+20 | CD14 | HLA-DR | CD45 |
| surf | TIM-3 | CXCR3 | CD4 | CCR6 | CD57 | CD8 | CD45 | PD1 | CD3 |
| surf | CD45 | CD24 | CD19 | IgD | CD38 | CD20 | CD27 |  | CD3 |
| surf | CD45 | IgG2a | CD4 | CD19 | IgG2b | CD8 | IgG1 |  | CD3 |
| surf | CD45 | CTLA4 | CD4 | CD19 | LIR1 | CD8 | PD-L1 | PD1 | CD3 |
| intra | IgG2a | IgG2a | CD4 | IgG1 | IgG1 | IgG2a | CD45 |  | CD3 |
| intra | CD25 | FoxP3 | CD4 | CCR4 | CD127 | CD45RO | CD45 | HLA-DR | CD3 |
| intra | IgG1 | IgG2b | CD4 | CD56 | CD19 | CD8 | CD45 |  | CD3 |
| intra | CD3ζ | perforin | CD4 | CD56 | CD19 | CD8 | CD45 |  | CD3 |
| intra |  | CTLA4 | CD4 |  | CD19 | CD8 | CD45 |  | CD3 |
| # | CD3 | CD16+56 | CD45 | CD4 | CD19 | CD8 |  |  |  |

**Supplementary Table 2.** Patients´ characteristics of the total and immune-monitored GeparNuevo trial

| parameter | category | no of analyzed patients (% of total) | | | | p (win)^e^ |
| --- | --- | --- | --- | --- | --- | --- |
|  |  | total trial ^a^ | total monitored ^b^ | total trial window ^c^ | monitored window ^d^ |  |
| patients | n | 174 | 120 | 117 | 63 |  |
| age | <30 | 10 (5.7%) | 5 (4.2%) | 8 (6.8%) | 3 (4.8%) | 0.8484 |
|  | 30 – 40 | 37 (21.3%) | 25 (20.8%) | 22 (18.8%) | 10 (15.9%) |  |
|  | 40 – 50 | 40 (23.0%) | 27 (22.5%) | 31 (26.5%) | 18 (28.6%) |  |
|  | 50 – 60 | 57 (32.8%) | 41 (34.2%) | 38 (32.5%) | 22 (34.9%) |  |
|  | 60 – 70 | 28 (16.1%) | 21 (17.5%) | 16 (13.7%) | 9 (14.3%) |  |
|  | ≥70 | 2 (1.1%) | 1 (0.8%) | 2 (1.7%) | 1 (1.6%) |  |
| grading | G1 | 0 (0.0%) | 0 (0.0%) | 0 (0.0%) | 0 (0.0%) | 1.0000 |
|  | G2 | 29 (16.7%) | 22 (18.3%) | 15 (12.8%) | 8 (12.7%) |  |
|  | G3 | 145 (83.3%) | 98 (81.7%) | 102 (87.2%) | 55 (87.3%) |  |
| nodal status | cN0 | 120 (69.0%) | 87 (72.5%) | 78 (66.7%) | 45 (71.4%) | 0.2133 |
|  | cN1 | 42 (24.1%) | 24 (20.0%) | 29 (24.8%) | 11 (17.5%) |  |
|  | cN2 | 8 (4.6%) | 6 (5.0%) | 7 (6.0%) | 5 (7.9%) |  |
|  | cN3 | 4 (2.3%) | 3 (2.5%) | 3 (2.6%) | 2 (3.2%) |  |
| tumor size | cT1 | 78 (44.8%) | 55 (45.8%) | 49 (41.9%) | 26 (41.3%) | 0.9134 |
|  | cT2 | 86 (49.4%) | 60 (50.0%) | 59 (50.4%) | 33 (52.4%) |  |
|  | cT3 | 6 (3.4%) | 3 (2.5%) | 5 (4.3%) | 2 (3.2%) |  |
|  | cT4 | 4 (2.3%) | 2 (1.7%) | 4 (3.4%) | 2 (3.2%) |  |
| TIL | low (0-10%) | 66 (37.9%) | 44 (36.7%) | 41 (35.0%) | 19 (30.2%) | 0.1428 |
|  | intermediate (11-59%) | 83 (47.7%) | 61 (50.8%) | 59 (50.4%) | 37 (58.7%) |  |
|  | high (≥ 60%) | 25 (14.4%) | 15 (12.5%) | 17 (14.5%) | 7 (11.1%) |  |
| stage | IIa and higher | 113 (64.9%) | 74 (61.7%) | 80 (68.4%) | 41 (65.1%) | 0.4322 |
| window treatment | present | 117 (67.2%) | 63 (52.5%) |  |  |  |

^a-d^:the GPN trial included a total of 174 patients (^a^), of which only 120 underwent blood immunomonitoring (^b^). Relative to treatment within the GPN total trial, 117 patients had the window treatment with durvalumab (^c^) and of those only 63 belonged to the patients with blood immunomonitoring (^d^).

^e^: p values compare the window patients of the total trial and those with blood immunomonitoring

**Supplementary Table 3.** Blood markers changing differently along treatment between the durvalumab and the placebo arm of the GeparNuevo trial

| Time point | Biomarker^a^ | n= | median of delta^b^ to T1 | | p value |
| --- | --- | --- | --- | --- | --- |
|  |  |  | durvalumab | placebo |  |
| T2 | # leukocytes | 49 | -0.176 | 0.101 | 0.025 |
|  | # granulocytes | 49 | -0.237 | 0.131 | 0.045 |
|  | # B cell | 49 | -0.430 | -0.090 | 0.034 |
|  | % B cell CD20^neg^ | 49 | 0.003 | -0.015 | 0.021 |
|  | CD8^+^ T CD45RO CCR4^neg^ | 49 | -0.300 | 7.750 | 0.012 |
|  | CD8^+^ T CXCR3^neg^ CCR6^+^ | 49 | 0.500 | -0.900 | 0.047 |
|  | % mDC | 49 | 0.136 | -0.245 | 0.050 |
| T3 | ratio # G/L | 54 | 0.350 | -0.267 | 0.034 |
|  | % T cell | 56 | 2.465 | -0.627 | 0.012 |
|  | CD4^+^ Tem | 55 | -5.000 | -0.950 | 0.022 |
|  | CD4^+^ T naïve | 55 | 8.100 | 2.900 | 0.046 |
|  | CD4^+^ T CD45RA | 56 | 8.473 | 2.700 | 0.041 |
|  | CD4^+^ T CD45RA HLA-DR^neg^ | 56 | 6.950 | 1.900 | 0.016 |
|  | CD8^+^ T CD28^neg^ CD56^neg^ | 55 | -0.675 | -1.900 | 0.042 |
|  | CD8^+^ T CD3ζ^+^ Perf^+^ | 23 | 6.100 | -4.650 | 0.015 |
|  | CD8^+^ T Perf^+^ | 23 | 7.500 | -4.650 | 0.031 |
|  | γδ T CD28^+^ CD56^+^ | 55 | 0.100 | 2.000 | 0.041 |
| T4 | # leukocytes | 58 | -0.045 | -0.341 | 0.004 |
|  | # granulocytes | 57 | 0.213 | -0.236 | 0.016 |
|  | ratio # G/L | 57 | 1.184 | 0.565 | 0.013 |
|  | CD4^+^ T CD45RA CD38^+^ | 59 | -11.100 | -4.720 | <0.001 |
|  | CD8^+^ T CD16^+^ CD28^neg^ | 59 | 2.595 | 0.600 | 0.031 |
|  | CD8^+^ T CD45RA CCR4^neg^ | 57 | -4.300 | -10.550 | 0.032 |
|  | % mDC | 57 | 0.523 | 0.072 | 0.039 |

^a^: #: cell counts per microliter blood; %: frequency within PBMC; no symbol: frequencies within the immune cell subsets (i.e CD4^+^ T, CD8^+^ T, γδ T); ^b^: for # is the delta of log2-transformed data; p value from a two-sided Wilcoxon test

**
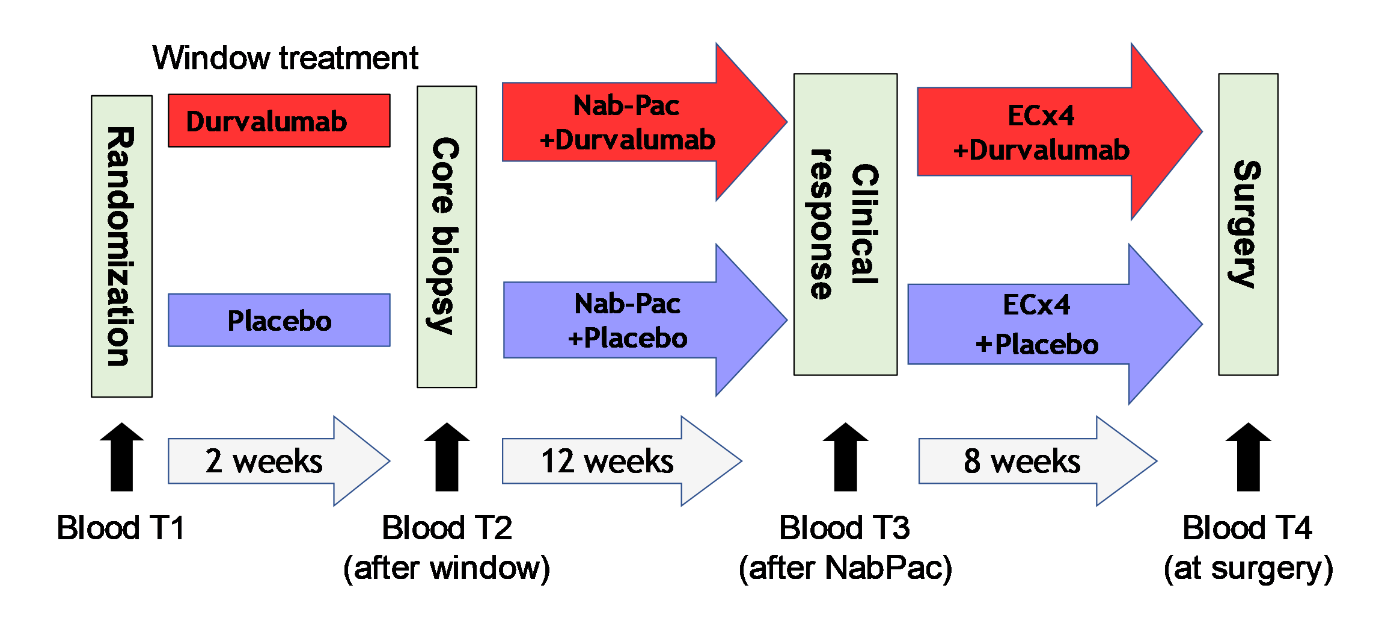
**

**Supplementary Figure 1.** Scheme of the GeparNuevo trial

TNBC patients were randomized to the durvalumab or placebo arm and underwent or not a window pretreatment of durvalumab or placebo before starting with NAC based on nanoparticle-bound paclitaxel (Nab-Pac) followed by epirubicin and cyclophosphamide (EC). At 4 different time point during treatment blood samples were collected in EDTA monovettes for immunomonitoring, which was performed within 24 h from blood draw.

**
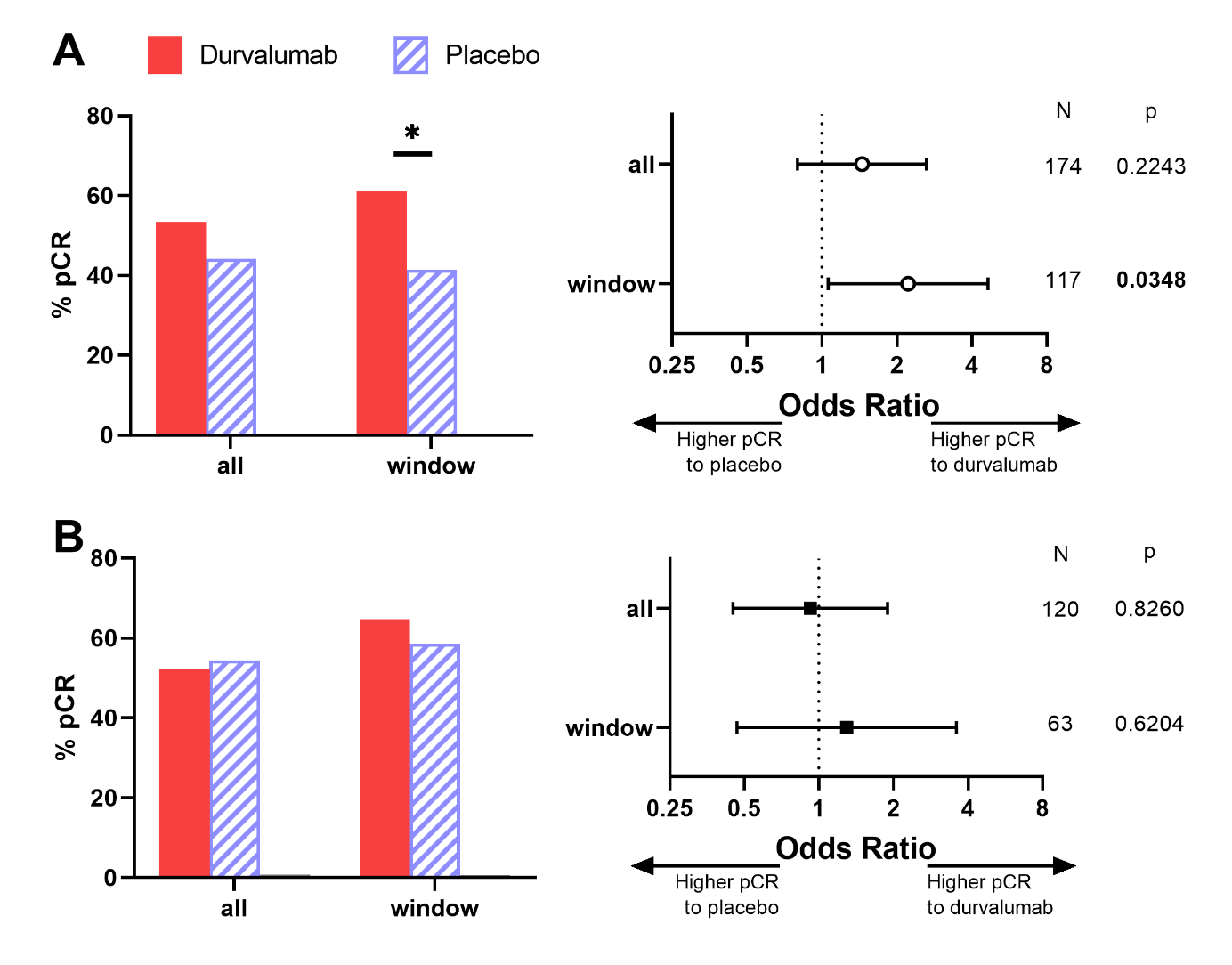
**

**Supplementary Figure 2.** Clinical outcome of the different sub-cohorts of the GeparNuevo trial

The frequencies of pathological complete response (pCR, *left*) as well as the OR (*right*) for the complete GeparNuevo cohort (A) and for the sub-cohort for which blood samples underwent immune-monitoring (B) are shown. The outcome of patients that received the window pre-treatment with durvalumab are also shown. According to the GeparNuevo protocol, pCR was defined as ypT0ypN0. *, significant difference between the treatment arms; N, number of patients; p, Wald p-value for odds ratio.

**
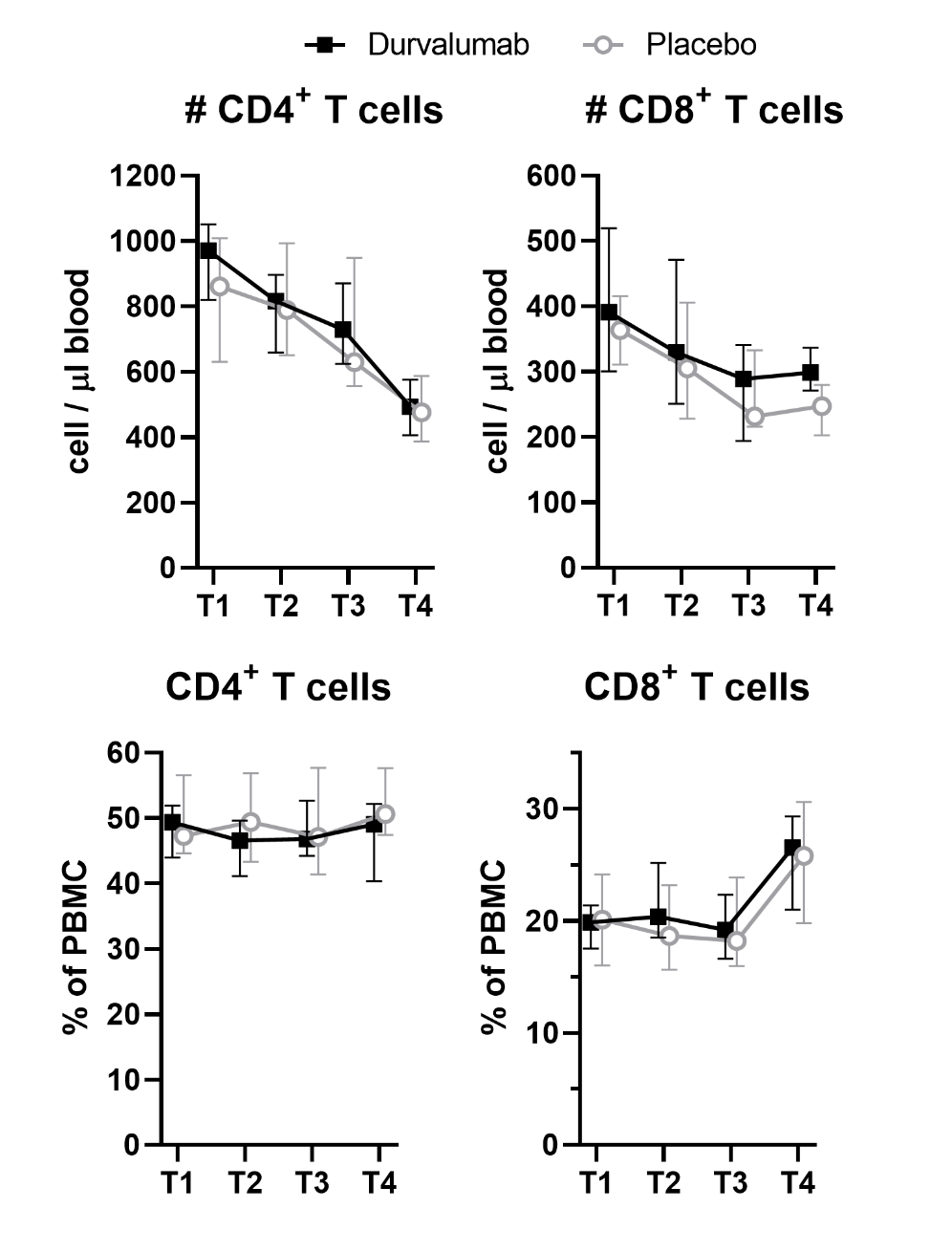
**

**Supplementary Figure 3.** Absolute cell counts and frequencies of CD4^+^ and CD8^+^ T cells during treatment

The absolute cell counts in the blood (*top*) as well as the frequencies within the PBMC (*bottom*) of CD4^+^ (*left*) and CD8^+^ T cells (*right*) during treatment are shown as median ± 95% CI.


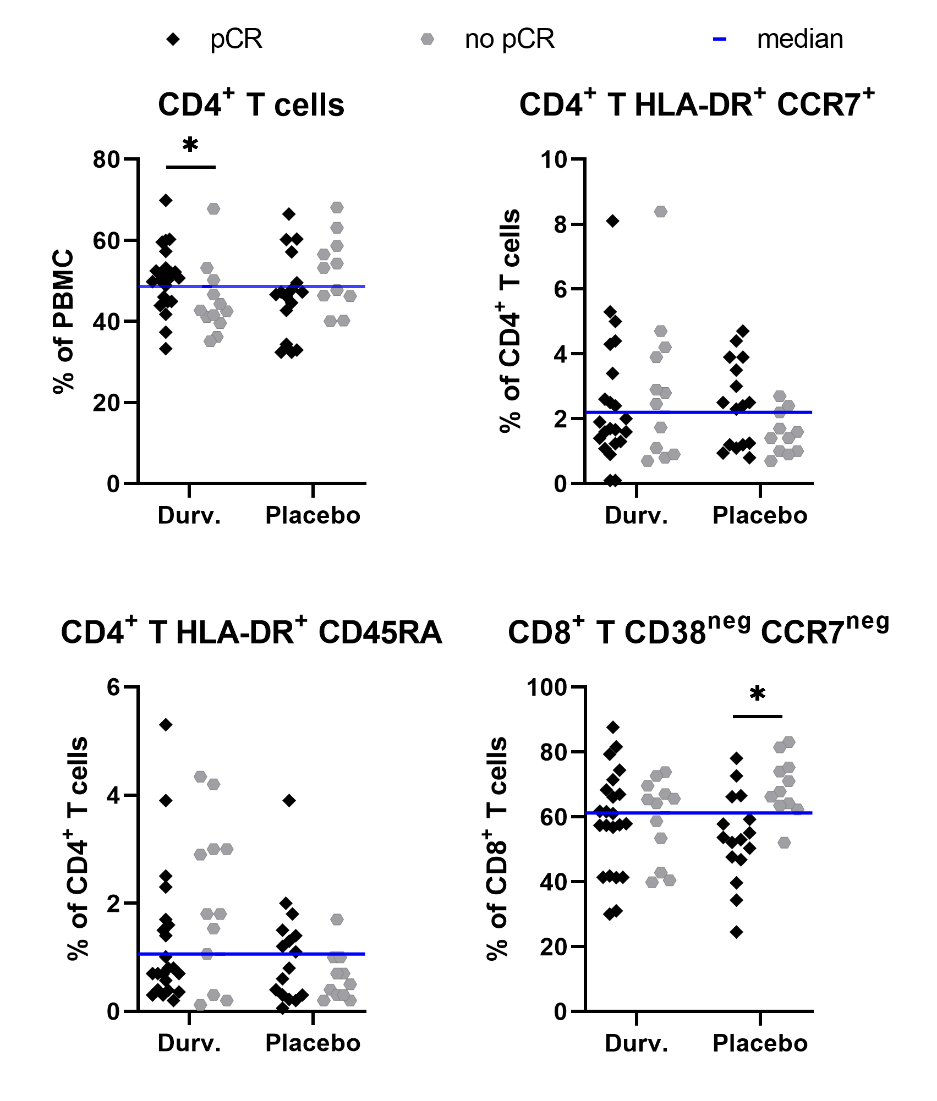


**Supplementary Figure 4.** Biomarkers at recruitment

The individual values at recruitment for the markers from Figure 2 are shown for the durvalumab and placebo patients with or without pCR. The blue lines correspond to the median value used for dichotomization. * p < 0.05 in the Wilcoxon test.


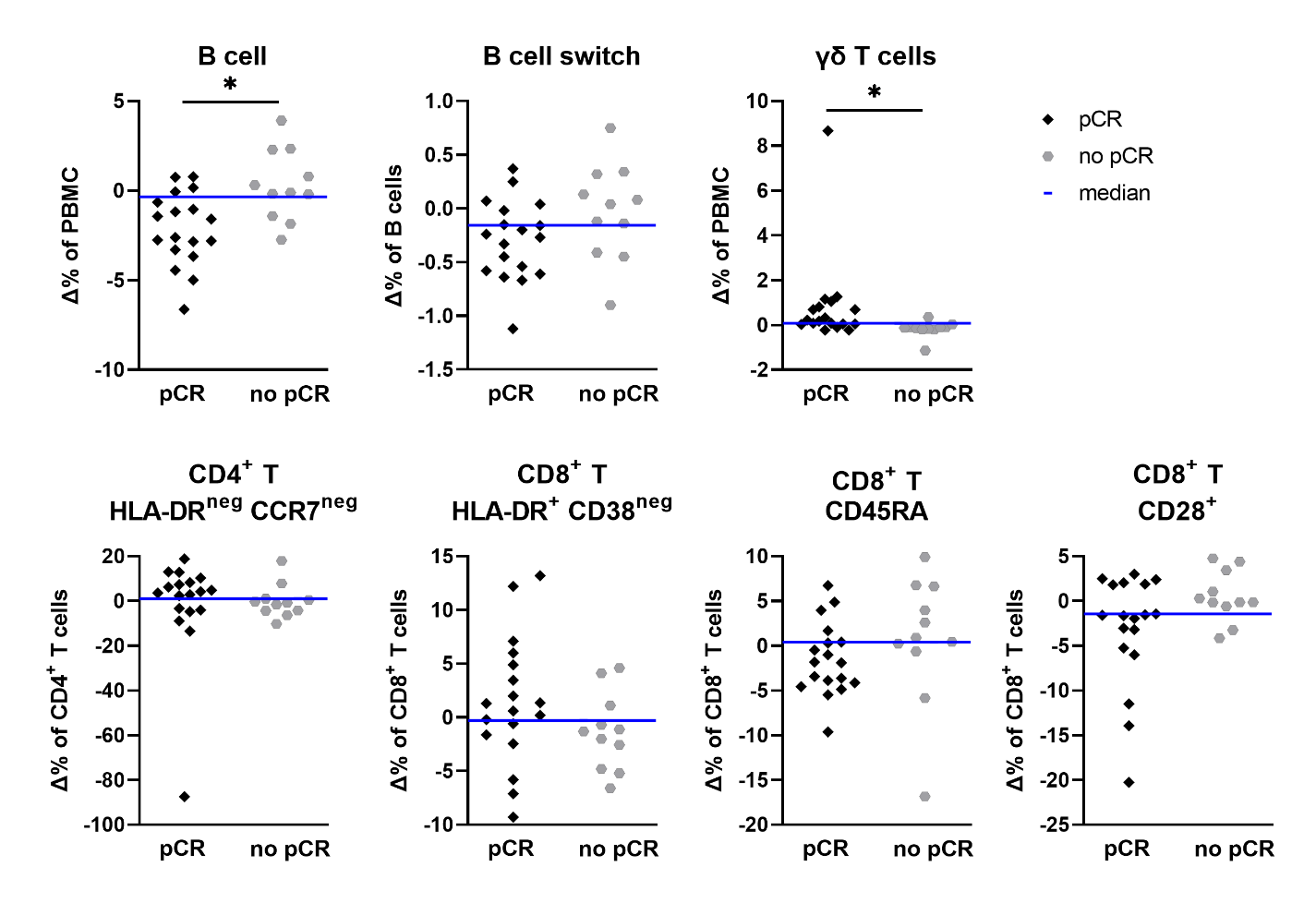


**Supplementary Figure 5.** Biomarkers after the window treatment with durvalumab

Shown are the delta between the frequencies (Δ%) at T2 and T1 of the markers from Figure 3A for each individual patient. The blue lines represent the median of the delta used for dichotomization. * p<0.05 in the Wilcoxon test.


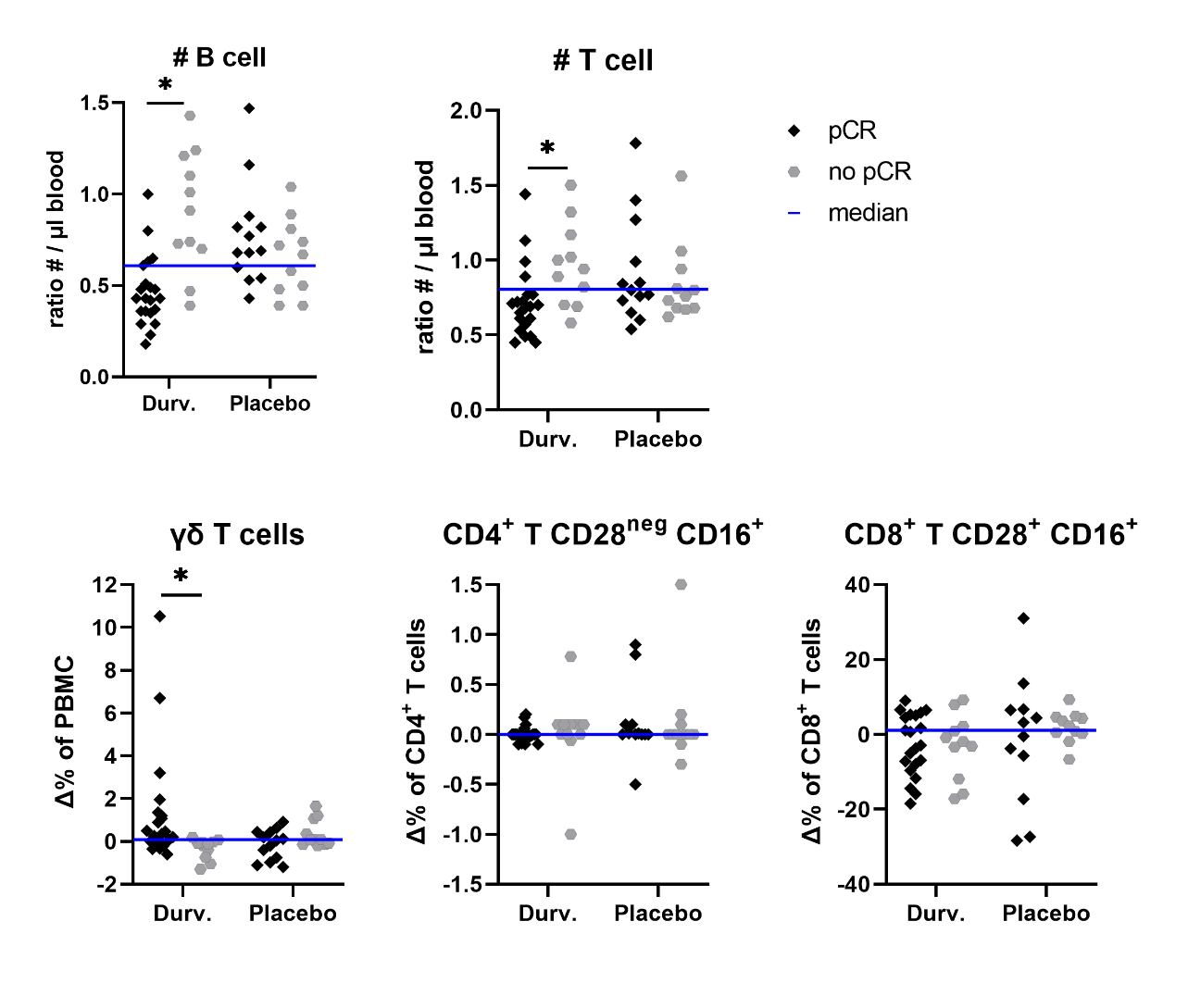


**Supplementary Figure 6.** Biomarkers after Nab-Pac

The ratio between T3 and T1 of the absolute number of cell per µl blood (ratio #) or the delta of the frequencies (Δ%) within the indicated populations for the markers from Figure 3B are shown for each individual patients of the durvalumab and the placebo arm grouped by pCR status. The blue lines represent the medians used as cutoff for dichotomization. * p<0.05 in the Wilcoxon test.


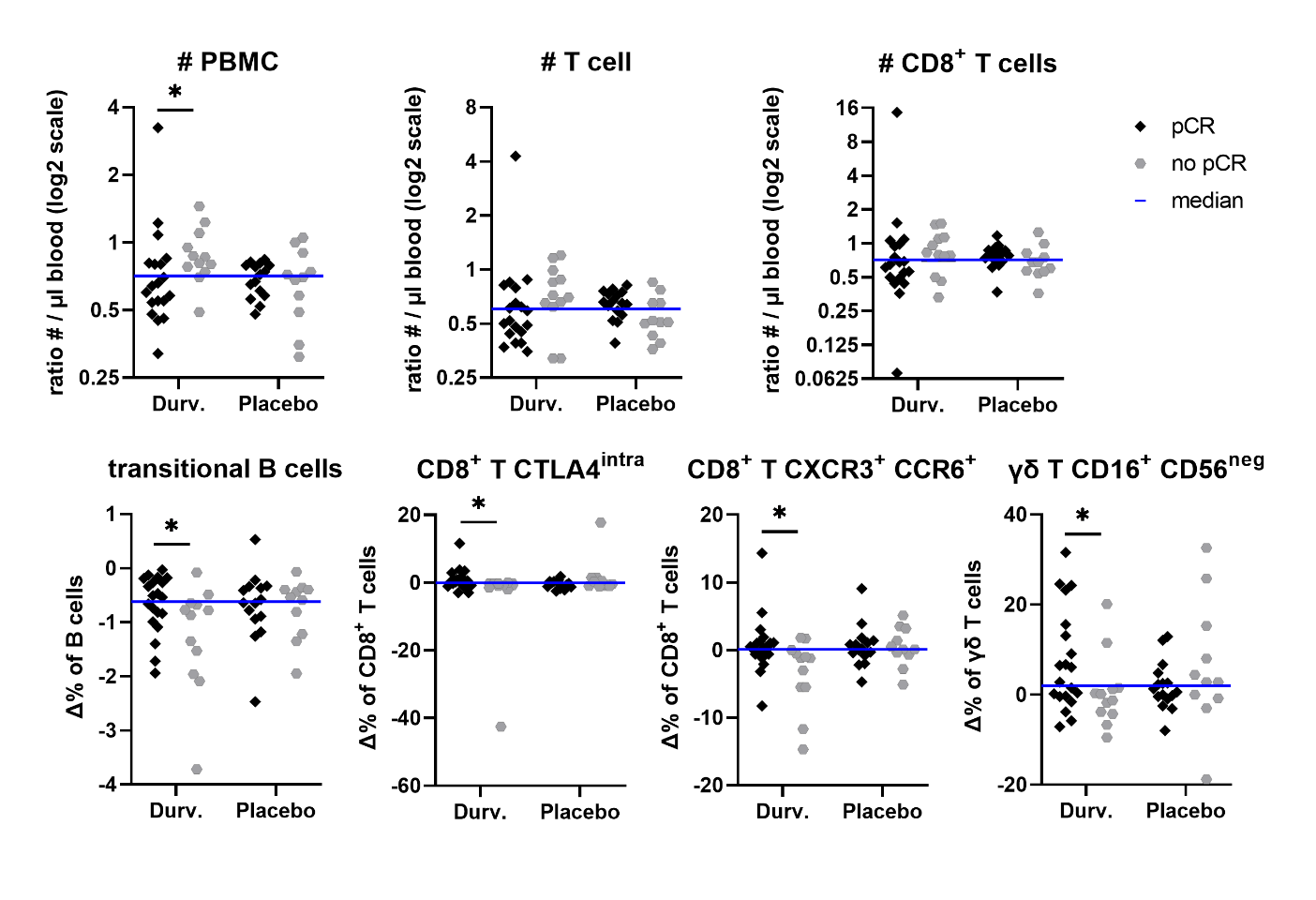


**Supplementary Figure 7.** Biomarkers at surgery

The ratio between the absolute number of cell (ratio #) per µl blood and the delta between the frequencies (Δ%) at T4 and T1 for the significant markers from figure 3C are shown for each individual patients from the durvalumab and the placebo arm in relation to their clinical outcome. The blue lines represent the median delta used for dichotomization. * p<0.05 in the Wilcoxon test.
